# Arthrofibrosis risk factors after anterior cruciate ligament reconstruction

**DOI:** 10.3389/fspor.2023.1264150

**Published:** 2023-10-11

**Authors:** Marc Dauty, Pierre Menu, Jérôme Grondin, Vincent Crenn, Pauline Daley, Alban Fouasson-Chailloux

**Affiliations:** ^1^ Service de Médecine Physique et Réadaptation Locomotrice et Respiratoire, CHU Nantes, Nantes Université, Nantes, France; ^2^ Service de Médecine du Sport, CHU Nantes, Nantes Université, Nantes, France; ^3^ Institut Régional de Médecine du Sport (IRMS), Nantes, France; ^4^ Inserm, UMR 1229, RMeS, Regenerative Medicine and Skeleton, ONIRIS, Nantes Université, Nantes, France; ^5^ Clinique Chirugicale Othopédique et Traumatologique, CHU Nantes, Nantes Université, Nantes, France

**Keywords:** stiffness, knee, surgery, rehabilitation, sport

## Abstract

**Introduction:**

Knee arthrofibrosis is a disabling complication after anterior cruciate ligament reconstruction (ACLr). Different risk factors have been studied but are still controversial because of a diagnosis made only during reoperations for the treatment of the stiffness, which underestimates the occurrence rate. We aimed to confirm risk factors of arthrofibrosis after ACLr in case of clinically made diagnoses.

**Methods:**

Ninety-two athletes with clinically diagnosed arthrofibrosis, complicating a primary ACLr, were compared to 482 athletes with ACLr without any complications. Usually considered risk factors were studied: age under 18, female, Body Mass Index (BMI ≥ 25), high sport level, time from ACL injury to ACLr < 1 month, Bone-Patella-Tendon-Bone surgical procedure (BPTB), meniscal repair, and intensive rehabilitation. Binary logistic regression was carried out to confirm or refute these risk factors.

**Results:**

Female, time from ACL injury to ACLr < 1 month, BPTB procedure, meniscal repair, and BMI ≥ 25 were not confirmed as risk factors. Previous competitive sport level assessed by Tegner score was the only risk factor identified, OR: 3.56 (95%IC: 2.20–5.75; p = 0.0001). Age < 18, OR: 0.40 (95%IC: 0.19–0.84; *p* = 0.015) and inpatient rehabilitation program, OR: 0.28 (95%IC: 0.17–0.47; *p* = 0.0001), were protective factors.

**Discussion:**

Competitive athletes are at risk of arthrofibrosis after ACLr and should benefit from protective inpatient rehabilitation program.

## Introduction

Anterior cruciate ligament reconstruction (ACLr) aims to restore a stable and painless knee with full range of motion. However, post-operative recovery may be complicated by knee pain associated to a limitation of range of motion, known as arthrofibrosis (1). The diagnosis can be difficult to make because isolated loss of knee extension can be explained by a technical error concerning the tibial graft placement (too anterior or too lateral), a high graft tension or a cyclops syndrome (2, 3). However, the combination of a limitation of knee flexion and extension is suggestive of arthrofibrosis (3, 4). Arthrofibrosis corresponds to a joint and peri-articular invasion of fibrous tissue responsible for joint ankylosis (5). Several types have been described according to the loss of knee range of motion and patella mobility (6). Type 3 arthrofibrosis corresponds to a defect of extension of more than 10 degrees and a defect of flexion of at least 25 degrees, with limited patella mobility also described as « infrapatellar contracture syndrome » (7). A large loss of isokinetic knee strength is associated with type 3 arthrofibrosis, and requires more than 12 months to improve (1, 6, 8). These functional deficiencies may explain why this complication is devastating regarding daily and sports activities (1, 8–10).

Due to an incidence ranging from 4 to 38% (11) and the consequences of this complication, several risk factors have been examined. A genetic predisposition has been described with the presence of HLA-Cw*07 and 08 alleles (12). Age < 18, female, time from ACL injury to ACLr < 1 month, Bone-Patellar-Tendon-Bone procedure, concomitant meniscal repair, intensive rehabilitation or prolonged immobilization were identified as possible risk factors (3, 9, 10, 13–16). Yet, these risk factors remain controversial because they have been studied in populations surgically treated for knee stiffness (10, 11, 14–16). The purpose of this retrospective study was to identify possible risk factors associated with clinically-diagnosed arthrofibrosis after ACLr, in patients who presented a stage 3 arthrofibrosis compared with patients without any complications at 4 postoperative months.

## Method

### Study design

This is a retrospective cohort study of 574 athletes with primary ACLr using Bone-Patella-Tendon-Bone (BPTB) or Hamstring (H) procedure, with or without concomitant meniscal repair.

### Population

Ninety-two patients operated for primary ACLr, were classified with type 3 arthrofibrosis at 4 post-operative months. Diagnosis was based on limitation in knee range of motion, with a loss of extension ≥10° and a loss of knee flexion ≥25° (6). Lysholm knee function score and isokinetic muscle knee strength at 60°/s (Limb Symmetry Index between the operated knee and the healthy knee) were also measured at 4 months (17, 18), due to their known decrease in case of arthrofibrosis. The number of rehabilitation sessions performed from ACLr to the 4th post-operative month was also reported, with the hypothesis that more rehabilitation sessions were prescribed to recover the knee range of motion in case of arthrofibrosis. These patients with arthrofibrosis were compared to 482 patients with ACLr who did not have any complications at 4 postoperative months. Exclusion criteria included patients with ACLr who had presented after surgery an infection, an anterior or posterior knee pain without knee range of motion limitation, a Cyclops syndrome or a complex regional pain syndrome were excluded (3).

All patients, recruited from 2012 to 2022 by an independent sport physician, had been operated by 15 different surgeons regularly performing ACLr procedures. Early post-operative rehabilitation involved inpatient or outpatient management on the principle of accelerated rehabilitation program (early full weight-bearing with crutches, early passive and active knee extension) (19, 20). The choice of inpatient rehabilitation (3 different rehabilitation centers) or outpatient rehabilitation with a physiotherapist was made by the patients before ACL surgery. Inpatient rehabilitation involved fulltime hospitalization for 3 weeks, with two physiotherapy sessions per day initially, followed by an additional daily session of adapted physical activity. The outpatient rehabilitation consisted of 30 to 40 sessions of physiotherapy, divided into 3 sessions per week. No supplement financial cost existed according to the two rehabilitation procedures.

All patients provided written consent, and the study was approved by the local ethics committee. The study followed the declaration of Helsinki (21).

### Outcomes variables

Studied arthrofibrosis risk factors were: age < 18, female, time from ACL injury to ACLr < 1 month, BPTB procedure, concomitant meniscal repair, “intensive rehabilitation” (that is to say a number of early post-operative sessions including 10 sessions a week from the 1st to the 3rd-4th week for inpatient rehabilitation in a rehabilitation sport center). The number of post-operative rehabilitation sessions at 4 post-operative months were reported. Prolonged knee immobilization was not considered a risk factor because all the patients had received accelerated rehabilitation (19, 20).

Other parameters have also been investigated as potential risk factors. Body Mass Index (BMI) was considered if it was greater than 25 kg/cm^2^ because full-support walking was permitted after ACLr (22). The level of sport before ACL injury ≥7 according to the Tegner activity scale (23) was also considered as a potential risk factor based on the assumption that athletes who practice a pivot and contact sport in competition want to return to sport faster. The Tegner activity scale ≥7 corresponds to competitive sports such as racquet sports, down-hill skiing, soccer, football, rugby, ice hockey, gymnastics, basketball, and we have added handball at a national competitive level (17).

### Statistical analysis

Statistical analysis was performed using the SPSS 23.0® software package [(Armonk, NY, USA)]. The quantitative variables were expressed by average and standard deviations. The categorical variables were expressed by median, maximum and minimum values, or frequency. The normality of the tested parameters was assessed by a Kolmogorov-Smirnov test. Univariate analysis (independent t-tests) and *χ*^2^ tests were used to compare quantitative and qualitative data of the arthrofibrosis and control ACLr groups. Results were considered significant at *p* < 0.05.

The effect of each risk factor was tested separately as categorical variable (under or upper the cutoff) (24). The prediction for the group with arthrofibrosis was assessed using binary ascendant logistic Wald's regression (inclusion probability ≤ 0.10). Logistic regression function predicts a logit transformation of the probability of arthrofibrosis, odds = probability/(1-probability) (25). We included in the binary logistic regression model, the exposure factors from the univariate analysis with a *p* < 0.20. In this study, the probability was the occurrence of arthrofibrosis in the ACLr group.

## Results

**Figure 1:**
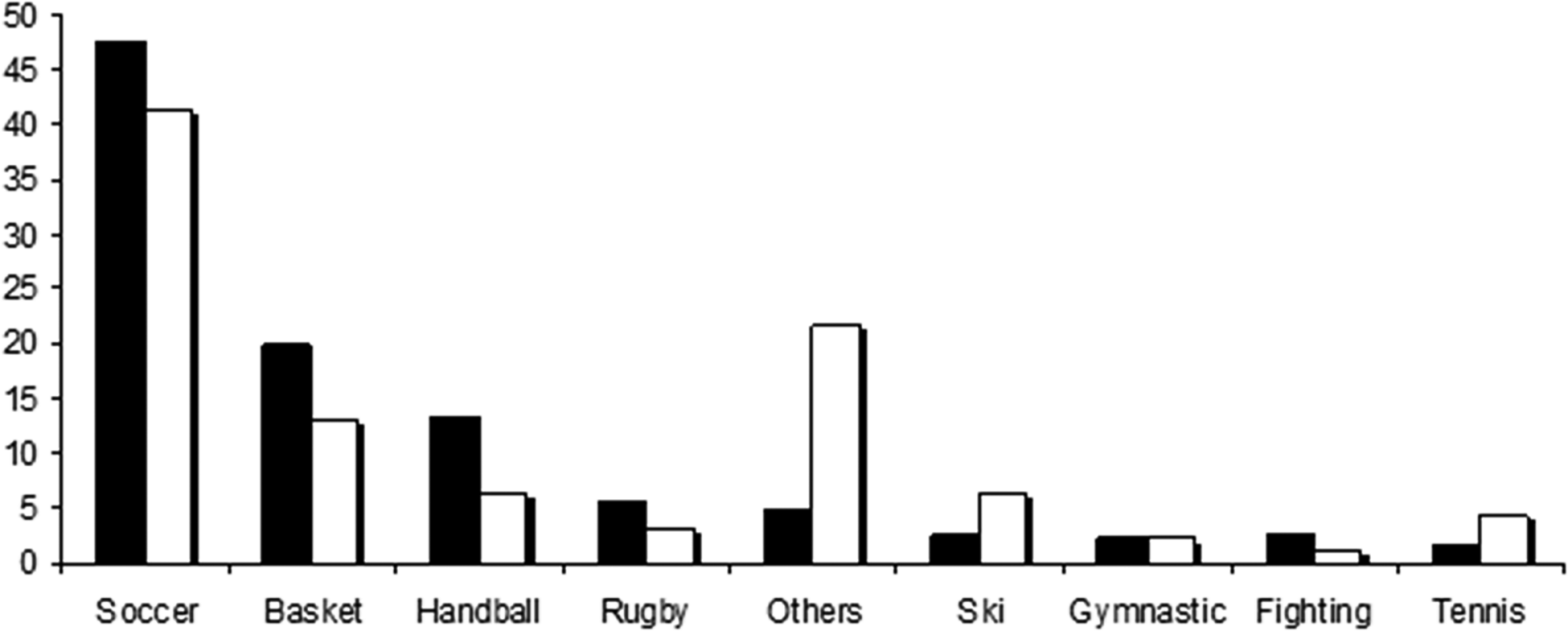
Frequency (%) of sport practice in arthrofibrosis and control ACLr groups. The group with arthrofibrosis after ACLr is in white, the group without arthrofibrosis is in black.

Both arthrofibrosis and control ACLr groups were comparable for anthropometric parameters (weight, height and BMI) (Table 1). The arthrofibrosis group was 4 years older and had performed 9 more rehabilitation sessions than the control group at 4 post-operative months. The arthrofibrosis group had, as expected, a functional Lyscholm knee score and an isokinetic knee strength (LSI) significantly smaller than the control group (Table 1). The practiced sports are described in Figure 1. The arthrofibrosis rate ranged from 10.5 to 16% according to the surgeons, without statistical difference.

**Table 1:** Comparison between the group with arthrofibrosis and the control group at 4 months after ACL reconstruction (student *t*-test).

	Arthrofibrosis group (*n* = 92)	Control group (*n* = 482)	*P*
Age (years)	28.0 ± 8.0	24.0 ± 6.0	0.0001
	[15–45]	[13–52]	
Weight (Kg)	70.0 ± 12.0	72.0 ± 12.0	0.24
	[50–115]	[43–145]	
Height (cm)	169.0 ± 7.0	173.0 ± 9.0	0.10
	[156–185]	[150–190]	
BMI (Kg/cm^2^)	22.5 ± 2.2	23.0 ± 3.4	0.43
	[19–28]	[17–37]	
Q60 LSI (%)	40 ± 14	69 ± 15	0.0001
	[7–78]	[46–105]	
H60 LSI (%)	65 ± 14	87 ± 13	0.0001
	[27–95]	[51–114]	
Lysholm score (Points / 100)	83 ± 9	96 ± 7	0.0001
	[57–92]	[76–100]	
Physical sessions (*n*)	45 ± 21	36 ± 15	0.0001
	[20–100]	[0–40]	

BMI, Body Mass Index; Q60 LSI, Quadriceps Limb Symmetry Index at 60°/s; H60 LSI, Hamstring Limb Symmetry Index at 60°/s. Physical sessions: number of post-operative physical sessions carried out from ACLr to 4 months post-surgery.

The frequency of parameters considered as arthrofibrosis risk factors were not different between the two groups, excepted for the age < 18, the in- or outpatient rehabilitation and the sport level (Table 2). However, the patients with a sport level ≥ 7 and those under 18 years old had significantly more inpatient management (60.1% vs. 39.9%; *p* < 0.01, and 24.1% vs. 13.5%; *p* = 0.005, respectively).

**Table 2:** Comparison of risk factors the group with arthrofibrosis and the control group after ACL reconstruction (*χ*^2^ test).

	Arthrofibrosis group (*n* = 92)	Control group (*n* = 482)	*p*
Age < 18 years	10 (10.9%)	111 (23%)	0.0008
Age ≥ 18 years	82 (89.1%)	371 (77%)	
Male	58 (63%)	345 (71.6%)	0.10
Female	34 (37%)	137 (28.4%)	
BMI < 25 Kg/cm^2^	75 (81.5%)	409 (84.9%)	0.76
BMI ≥ 25 Kg/cm^2^	17 (18.5%)	73 (15.1%)	
Duration < 1 month	14 (15.2%)	81 (16.8%)	0.76
Duration ≥ 1 month	78 (84.8%)	401 (83.2%)	
H procedure	56 (60.9%)	332 (68.9%)	0.14
BPTB procedure	36 (39.1%)	150 (31.1%)	
No meniscus repair	72 (78.3%)	349 (72.4%)	0.30
Meniscus repair	20 (21.7%)	133 (27.6%)	
Inpatient management	29 (31.5%)	297 (61.6%)	0.0001
Outpatient management	63 (68.5%)	185 (38.4%)	
Sport level < 7	51 (55.4%)	110 (23.2%)	0.0001
Sport level ≥ 7	41 (44.6%)	370 (76.8%)	

BMI, Body Mass Index; Duration, Duration between ACL injury and reconstruction; Physical sessions, number of post-operative physical sessions carried out at 4 months post-surgery. H and BPTB procedure, Hamstring or Bone-Patellar-Tendon-Bone procedure.

The sport level ≥ 7 was a risk factor, OR = 3.13 (95%IC: 2.16–4.53), while age < 18, OR = 0.45 (95%IC: 0.24–0.85) and the inpatient rehabilitation procedure, OR = 0.35 (95%IC: 0.23–0.52) were protective factors according to an OR exposure < 1 (Table 3). The best model was able to correctly classify 84% of the patients with arthrofibrosis, which included 3 different parameters, age < 18, inpatient rehabilitation program and sport level ≥ 7 (Table 4). The classification accuracy of the final model could predict 36% of the outcome “arthrofibrosis”. The data fitted the model well (Hosmer-Lemershow test: *p* = 0.21) and the model was well adjusted (Cox and Snell, and Nagelkerke R-squares of 0.10 and 0.18, respectively).

**Table 3:** Exposure factors of arthrofibrosis group (univariate analysis).

	Exposure odd for arthrofibrosis	95% IC
Age < 18 years	0.45	0.24–0.85
Female	0.72	0.49–1.06
BMI ≥ 25 Kg/cm^2^	0.87	0.39–1.93
Duration < 1 month	1.10	0.65–1.86
BPTB procedure	0.74	0.51–1.09
Meniscus repair	1.30	0.82–2.07
Inpatient management	0.35	0.23–0.52
Sport level ≥ 7	3.13	2.16–4.53

BMI, Body Mass Index; Duration, Duration between ACL injury and reconstruction; BPTB procedure, Bone-Patellar-Tendon-Bone procedure.

**Table 4:** Arthrofibrosis model from risk and protective factors (binary logistic regression).

Factors		Beta	Wald	OR	95% IC	*p*
Protective	Inpatient rehabilitation	−1.24	24.1	0.28	0.17–0.47	0.0001
	Age < 18 years	−0.89	5.91	0.40	0.19–0.84	0.015
Risk	Sport level ≥ 7	1.27	27	3.56	2.20–5.75	0.0001

BPTB procedure, Bone-Patellar-Tendon-Bone procedure.

## Discussion

In this retrospective study, we have highlighted that previous competitive sport level assessed by Tegner score was the only identified risk factor for arthrofibrosis, and that age < 18 and inpatient rehabilitation program were protective factors. We also reported that female sex, the association with a meniscus repair, the time from ACL injury to ACL reconstruction < 1 month, and the BPTB procedure had no impact on the occurrence of arthrofibrosis.

Despite the arthrofibrosis definition proposed by Shelbourne et al. in 1996, the clinical diagnosis of this knee complication is not always easy to make after ACLr, because of the postoperative knee swelling that can lead to pain associated with a loss of knee range of motion in extension and flexion (6, 26). At 4 post-operative months, the persistence of these clinical signs (in the absence of knee swelling) associated to a loss of knee function, a deep loss of isokinetic knee muscle strength and an increase of rehabilitation session number provide arguments in favor of knee arthrofibrosis (1, 4, 8).

The originality of our study was therefore to diagnose knee arthrofibrosis from clinical signs in a large cohort of patients, and not according to procedures for treatment of the stiffness (mobilization under anesthesia or surgical arthrolysis). Indeed, the surgical treatment for knee stiffness likely underestimates the number of arthrofibrosis due to undiagnosed cases or because some patients refuse surgical revision (3, 4, 9, 11).

We have also studied parameters according to the exposure odds ratio. To our knowledge, few studies have used this method to define the risk factors associated to arthrofibrosis (10, 14–16, 27). Nwachukwa at al. and Ouweleen et al. focused specifically on a pediatric population while Sanders et al. and Huleatt at al. studied an adult population (10, 14–16, 27).

In pediatric populations (mean age of 15), female sex, the association with a meniscus repair and the BPTB procedure have previously been identified as risk factors (14, 16). Yet, our population's age varied from 13 to 52, and therefore was very different from an exclusively pediatric population. Indeed, only 121 of our 574 ACLr patients (21%) were under 18 years old and the minimum age of our population was 13 years against 7 years for the study of Nwachukwa et al. (14). In addition, age below 18 represented a protective factor in our study (OR = 0.35 according to the multivariate model), probably due to the joint flexibility of children, which decreases the loss of knee range of motion.

In adults, Sanders et al. have shown from an epidemiological and historical observational cohort that female sex represented a risk factor, with a hazard ratio of 2.6 (10).Yet, in their study, age, the BPTB procedure, the meniscus repair and the time from ACL injury to ACLr < 1 month did not represent risk factors. Only 23 ACLr of their patients underwent a surgical revision for arthrofibrosis out of 1,355 ACLr patients (1.7%) (10). Huleatt et al. showed after multivariate analysis in adults that quadriceps tendon autograft procedures associated to concomitant meniscal repairs were independent risk factors (15). However, the authors included other risk factors such as knee infection and revision ACL reconstruction; parameters that we have not studied. Their incidence of arthrofibrosis was low, 4.5% in 2,424 ACLr according to the manipulations under anesthesia or lysis of adhesion (15).

The type of ACLr procedure is debatable as an arthrofibrosis risk factor. The BPTB surgical procedure and meniscus repair did not represent any risk factors in our study as already reported (10, 16, 27). In the same way, Huleatt et al. reported the quadriceps tendon autograft procedure as a risk factor but not the BPTB autograft procedure (15). Unfortunately, we have not studied the quadriceps tendon autograft. Mayr et al., in 2004, showed an association between arthrofibrosis and BPTB procedure, undoubtedly linked to the frequency of this type of procedures, which had been performed in 75.3% of ACLr. Only 8.5% of ACLr had benefited from a hamstring procedure (4). Cosgarera et al. showed that the meniscus procedure was not associated to arthrofibrosis (28).

In their literature analysis, Wang et al. only identified female sex as a potential risk factor of arthrofibrosis (27). Yet, the association between female sex and arthrofibrosis remains unknown (15, 27). Sanders et al. have evoked for females, a low tolerance to postoperative pain and a fear of a surgical revision in the event of knee joint range of motion loss (10). To date, these arguments do not seem admissible given the postoperative analgesic treatments and the conditions of surgical revision. However, a combination of social, psychosocial, musculoskeletal, and hormonal difference has been proposed (15, 27).

The time from ACL injury to ACLr < 1 month was not a risk factor as already reported by Sanders et al. (10, 15). Only comparative studies had shown this association, probably due to pre-operative knee irritation (swelling effusion, hyperthermia, loss of range of motion) at the time of surgery (4, 13). In fact, the time from ACL injury to ACLr does not represent a risk factor if the ACLr is performed on a painless knee, without swelling and with full range of motion (6, 9).

Since the existence of accelerated rehabilitation, the intensity of rehabilitation programs have been questioned to explain the occurrence of arthrofibrosis (19). Muscular retraining performed too early could be responsible for post-operative knee pain and inflammation, causing arthrofibrosis (3, 4). We tried to quantify aggressive rehabilitation according to the BMI (cutoff at 25 kg/cm^2^), the in- or outpatient rehabilitation program and the sport level (cutoff of the Tegner activity score ≥ 7). BMI ≥ 25 Kg/cm^2^ did not represent a risk factor for arthrofibrosis, while we might have thought that postoperative loading of an overweight ACLr patient could have been the cause of knee pain and swelling, explaining the arthrofibrosis occurrence. In our study, inpatient rehabilitation procedure represented a protective factor for arthrofibrosis (OR = 0.28), contrary to our hypothesis, possibly due to a rehabilitation carried out in 3 specialized sport rehabilitation centers. Indeed, the daily adapted supervision of the accelerated rehabilitation program by a physician specializing in rehabilitation may have reduced the knee swelling episodes, and made the knee painless and mobile. Undoubtedly, it contributed to avoiding arthrofibrosis.

High competitive sport level assessed by Tegner score ≥7 was the only risk factor reported in the present study. The hypothesis that sport issues could be the causes of overly intensive rehabilitation may have been confirmed, particularly if the patient had outpatient management. The patient too eager to return early to sport, and not sufficiently supervised by a physiotherapist, may have performed muscle strengthening exercises too prematurely compared to what his operated knee could tolerate (9).

Our model of arthrofibrosis in ACLr patients associated age < 18 and inpatient procedure as protective factors, and sport level ≥ 7 as a risk factor. According to these 3 factors, the inpatient procedure represents the only modifiable parameter before ACLr. Yet, all ACLr patients do not have the possibility of having access to this type of specialized and supervised rehabilitation programs. Patients' education, especially if they practice a pivotal contact sport in competition at a national level, should be improved in order to early recognize arthrofibrosis clinical signs and to avoid knee irritation (29). Early recognition of post-ACLr arthrofibrosis by the patient and the physiotherapist remains the key element to avoid knee range of motion loss (3).

This study has several limitations. Firstly, the diagnosis of arthrofibrosis was based on the old classification of Shelbourne et al. (6). Since then, a consensus has been developed with a more precise classification related to knee range of motion loss (26). This classification did not exist when our cohort was created, so it could not be used. Yet, the association of stage 3 arthrofibrosis, the isokinetic strength loss, the function loss, and the number of rehabilitation sessions carried out, may be sufficient to confirm arthrofibrosis diagnosis. Secondly, other risk factors have not been studied such as infection, ACL revision or primary ACL reconstruction with different grafts for instance (Quadriceps tendon, tibialis anterior tendon, allograft…) (15). In the same way, we did not assess the severity of the primary ACL injury. Yet, including these parameters would have increased the difficulty of understanding statistical association models.

Thirdly, risk factors represented only associated factors and not explanatory factors of arthrofibrosis. Actually, the pathological mechanisms of arthrofibrosis are better understood (30, 31). It is explained by a joint invasion of fibrous tissues responsible for a joint ankyloses. It is secondary to a fibroblastic and endothelial proliferation—a dense type I, II and IV collagen fibers formation depending on an overexpression of cytokines such as TGF-β, platelet-derived growth factor and fibroblastic growth factor. Fourthly, due to the retrospective design of the study, we did not assess the genetic component of arthrofibrosis, so our model was not able to include this potentially relevant risk factor. The treatment remains limited to rehabilitation program and inflammation control with the objective of recovering the knee range of motion without causing excessive TGF-β mutation which is at the origin of joint fibrosis (30). Knee surgery such as lysis of adhesions should be reserved for patients failing the rehabilitation treatment. Future treatments such as TGF-β or IL-1 antibodies may be promising (30). Finally, inpatient rehabilitation may appear unusual, but it is a local habit, which may be different in other countries. In our practice, inpatient rehabilitation remains always proposed to every patient without distinction.

## Conclusion

This study showed that the age < 18 and the use of a specialized rehabilitation center represented protective factors against arthrofibrosis, whereas the sport level assessed by Tegner score ≥ 7, was a risk factor. None of the other risk factors of arthrofibrosis cited in the literature have been confirmed. Because of few modifiable risk factors, patients with a sport level assessed by Tegner score ≥ 7 should benefit from an inpatient rehabilitation or at least a rehabilitation program supervised by expert physiotherapists specializing in ACLr to avoid a too aggressive rehabilitation for the operated knee.

## Data Availability

The raw data supporting the conclusions of this article will be made available by the authors, without undue reservation.
